# Experimental Researches Applied to Physiology and Pathology

**Published:** 1853-03

**Authors:** E. Brown-Séquard

**Affiliations:** Paris


### MEDICAL EXAMINER,

AND

### RECORD OF MEDICAL SCIENCE.

NEW SERIES.—NO. XCIX.—MARCH, 1853.

### ORIGINAL COMMUNICATIONS.

### *Experimental Researches applied to Physiology and Pathology.*

By E. Brown-Séquard, M. D., of Paris.

(Continued.)

#### XXII.—ON THE INCREASE OF ANIMAL HEAT AFTER INJURIES OF THE NERVOUS SYSTEM.

In another part of this series* I have endeavored to prove that the local increase of temperature following the section of the sympathetic nerve, is the result of paralysis of the blood-vessels. I will now relate some other cases in which a local increase of temperature takes place after various other injuries of the nervous system, and apparently in consequence of the same cause.

It was known, long ago, that an injury to the nervous system might be followed by a partial or even a general elevation of animal heat. Sir B. Brodie says,† Mr. Chossat has published an account of some experiments on animals, in which he found

* Medical Examiner, August 1853, p. 489.

† Medico-Chirurg. Transactions, 1837. Vol. xx., p. 132.

that the division of the superior portion of the spinal cord produced a remarkable evolution of animal heat, so that it was raised much above the natural standard. I have made experiments similar to those of Mr. Chossat, and have met with similar results. I have also seen several cases in which an accidental injury of the spinal cord has produced the same effect. The most remarkable of them was that of a man who was admitted into St. George's Hospital, in whom there was a forcible separation of the fifth and sixth cervical vertebræ, attended with an effusion of blood within the theca vertebralis, and laceration of the lower part of the cervical portion of the spinal cord. Respiration was performed by the diaphragm only, and, of course, in a very imperfect manner. The patient died at the end of twenty-two hours; and, for some time previous to his death, he breathed at very long intervals, the pulse being weak and the countenance livid. At last there were not more than five or six inspirations in a minute. Nevertheless, when the ball of a thermometer was placed between the scrotum and the thigh, the mercury rose to 111° of Fahrenheit's scale. Immediately after death, the temperature was examined in the same manner, and found to be still the same.

Brodie was mistaken as regards the experiments of Chossat. Instead of finding an increase in the animal heat after the section of the inferior portion of the spinal cord, Chossat found a considerable diminution in the temperature of dogs. But in two cases, where the spinal cord was divided at about the level of the last dorsal vertebra, in dogs, Chossat* found an increase in the animal heat. In one of these experiments, the increase was from 41°.1 to 41°.5 Cents., (105°.98 to 106°.7 Fahr.) In the other, the increase was from 41°.1 to 42°.9 Cs., (105°.98 to 109°.6.)

Dr. Macartney† found an increase in the temperature of parts paralyzed in consequence of the division of their nerve. H. Nasse,‡ who made many experiments on this subject, some-

Mèm. sur l' influence du syst. nerv. sur la chal. anim. Thèse de Paris. No. 126.—1820, p. 35. Exps. xxiii and xxiv.

† Treatise on Inflammation, 1838, p. 13.

‡ Vutersurchunger zur Physiol. und Pathol., 1839, v. ii., p. 190.

times observed an elevation in the temperature of the paralyzed parts after the division of the sciatic nerve, or after the partial destruction of the spinal cord.

In more than twenty experiments, I only once found an increase in the temperature of the leg of a guinea-pig, after the section of the sciatic nerve. This increase lasted about two or three days after the operation, and it was of two degrees Fahr.

After a complete transversal section of the spinal cord in the lumbar region, in birds and mammals, I found, repeatedly, an increase of one, two or three degrees Fahr. in the temperature of the paralyzed parts. I ascertained that it is not in consequence of an increase of the general temperature of the animal that such an increase exists. It is to be found only in the paralyzed parts.

I never found any increase of temperature after a complete transversal section of the spinal cord, either in the cervical or in the dorsal region.

After a section of a lateral half of the spinal cord, at the level of one of the three or four last dorsal vertebræ, I have almost constantly found an increase in the temperature of the posterior limb on the side of the section. The elevation varied from one to four degrees Fahr. On the contrary, there was a diminution of from one to five degrees Fahr. in the temperature of the other leg. In some cases, in consequence of the increase of temperature on one side and its diminution on the other side, I found a difference of six or seven degrees Fahr. in the temperature of the two limbs. It is very remarkable that, together with the increase of temperature in one limb, there is an augmentation of sensibility, and with the diminution of temperature in the other limb, there is also a diminution of sensibility.

Since the publication of the results of my experiments on the sympathetic nerve, I have performed them many other times, and I have found that the result is not so constant as Dr. A. Barnard and myself had admitted. In some rabbits there was no decided increase in the vascularization and in the temperature of the face. I ought to say that, in these cases, the two ears were already warm, and very vascular before the operation. I have found, also, that generally in very cold weather the extremity of the ear of rabbits, on the side of the section of the sympathetic nerve, remains cold.

From my experiments and from the observations and experiments of Brodie, Chossat, H. Nasse and Macartney, it results that the following opinion of Dr. Cl. Bernard is incorrect. He says: "It is known that injuries of the cerebro-spinal nervous system constantly produce a total or a partial diminution in the temperature of animals, either when a nerve has been divided or when the injury is made on the nervous centres."* He says also that an injury of the sympathetic nerve produces a very rapid increase of temperature; so that the sympathetic nerve and the cerebro-spinal nervous system are considered by Dr. Bernard as completely different, one from the other, as to the influence on animal heat when they are injured. The one should increase and the other diminish animal heat.

The truth is that these two effects—increase and diminution—may exist after an injury of either the sympathetic or the cerebro-spinal nervous system;† and, in both cases, the increase may exist, at first, and be followed by a diminution.

Before pointing out the co-existence of certain facts with the increase or diminution of animal heat, I think it necessary to establish a distinction between the cases of increase of animal heat after injuries of the nervous system.

In some cases (as those related by Brodie) there has been an increase of temperature above the natural standard of animal heat. These are very extraordinary and very rare cases, and it is not my intention to attempt to explain them here. In the cases, the degree of temperature, although increased in some other paralyzed parts, has not been above the normal degree of blood heat. This is the only kind of increase of animal heat that I have observed, and this I will attempt to explain.

I have found that,—*ceteris paribus*,—the more the arteries and capillaries are dilated, the higher is the degree of temperature. This law is proved by the following facts:

1st. In all the cases of paralysis (from whatever cause) where I have found a diminution in the degree of temperature

*Gaz. Medic. de Paris, Vol. 7, No. 14, p. 227.

† Vide: Chossat, *loco cit.*, pp. 41-46.

of a paralyzed part, the arteries and capillaries were evidently much contracted.

2d. In the cases where the temperature was normal, the blood-vessels were of their natural size.

3d. In the cases where the temperature was increased, I have constantly found the arteries and capillaries enlarged.

4th. In some cases, I have found the same changes occurring in the temperature and in the blood-vessels. The temperature at first was greater than usual and the blood-vessels dilated; afterwards both the temperature and blood-vessels became natural; and, at last, the temperature becoming lower than usual, the size of the blood-vessels became smaller.

I need not say that the changes occurring in paralyzed parts in accordance with the size of the blood-vessels were the results of the differences in the amount of blood passing in these parts.

Now, it will be asked how, in certain cases of palsy, the size of the blood-vessels is larger than usual, and smaller in other cases. I cannot explain how it is so, but I can assert that it is a fact.

From the facts and reasonings related in this article, I draw the following conclusions:

1st. An injury of the nervous system may produce in the parts, which then become paralyzed, either an increase or diminution of temperature.

2d. The sympathetic nerve and the cerebro-spinal nervous system appear not to be different one from the other, in this respect.

3d. The degree of temperature of paralyzed parts depends on the quantity of blood they receive; and this quantity varies according to the size of the arteries and capillaries of these parts.

4th. It is a fact, hitherto unexplained, that the arteries and capillaries may be either dilated, normal, or contracted in paralyzed parts.

XXIII.—CAUSE OF THE STOPPING OF THE HEART'S MOVEMENTS, PRODUCED BY AN EXCITATION OF THE MEDULLA OBLONGATA OR THE PAR VAGUM.

E. H. and E. Weber have discovered a singular fact, hitherto unexplained. When the par vagum or the medulla oblongata is excited by a powerful electro-magnetic current, in a living animal, the movements of the heart are suddenly stopped. This should be what is known for all motor nerves and muscles, if the cessation of the movements of the heart was the result of a permanent contraction. But the heart is not at all contracted, and, on the contrary, it remains perfectly placid.

This is entirely different from what we know to be the case for other muscles.

I have found that a violent mechanical excitation of the medulla oblongata produces also the same stopping of the heart's action.

Is the heart in a state of rest in consequence of a loss of its irritability or of an interruption of the excitation necessary to its action? The following fact proves that this second opinion is the right one. When the heart is stopped, every direct excitation upon it produces some beatings, and then, its irritability appears to be entire. The stopping, consequently, depends on the absence of excitation.

The cause exciting the heart to beat is in the blood contained in the capillaries of this organ, as I will try to prove in another article. Now, if we suppose that the galvanization of the par vagum produces a complete constriction of the capillaries of the heart, it is easy to understand why the heart is stopped: it is because the excitation cannot take place on account of the expulsion of the blood from the capillaries.

It will be asked on what ground we base the supposition that the capillaries are so contracted that they prevent entirely, or nearly so, the passage of the blood. I will answer:

1st. That it is known that a galvanization of certain nerves (and I have discovered that it is so with the capillaries of the face and ear when the sympathetic nerve is galvanized) may produce a considerable constriction of capillaries.

2d. That it is known that the nerves of the heart are distributed much more to its blood-vessels than to its muscular tissue.

3d. That, by our supposition, we place the fact of the stopping of the heart's movements among the well known facts, that an excitation of a molar nerve produces a contraction of the muscles to which it is distributed; and, therefore, we are not obliged to admit that an excitation of a nerve is able to produce directly either a contraction of or the cessation of existing contractions.

There is a practical consequence to be drawn from the fact that in the case of an excitation of the medulla oblongata, the stopping of the heart is not produced by a loss of irritability of this organ.

Many cases of syncope are produced by a stopping of the heart's movements in consequence of the influence of an emotion on the medulla oblongata. In these cases it would be of very great importance to excite directly the beatings of the heart, either by compression of the chest or by an application of galvanism.

We ought to say that galvanism applied directly to the heart increases its beatings instead of diminishing them.

### XXIV.—ON A SINGULAR DISTURBANCE IN THE VOLUNTARY MOVEMENTS, APPARENTLY PRODUCED BY AN ACTION OF ATMOSPHERIC AIR ON THE GRAY MATTER OF THE SPINAL CORD, IN BIRDS.

Some years ago I discovered that after the removal of a large quantity of the gray matter that exists in birds, on the posterior surface of the spinal cord, in the lumbar region, a great disturbance took place in the voluntary movements. I attributed this disturbance to the loss of gray matter. I have found, three or four months since, that the same disturbance existed in the voluntary movements after I had merely laid bare the gray matter, and immediately after it had been exposed to the action of air.

I am perfectly satisfied that it is not in consequence of a mechanical excitation of the spinal cord, accidentally produced during the operation of the removal of the bones and membranes, that this disorder takes place.

When the spinal cord is laid bare elsewhere than in the region of the lumbar enlargement—that is, in any place where the white substance covers completely the gray matter—there is no disturbance produced in the voluntary movements.

That disturbance very much resembles the so-called titubation which exists after either the removal of the cerebellum or the section of muscles of the posterior part of the neck. At each movement of progression, the animal tends to fall either forwards, backwards, or laterally. It does not fall completely, but is obliged, in order to avoid falling, to make use of its wings, its tail and its beak.

(To be continued.)

